# Functions and evolution of FAM111 serine proteases

**DOI:** 10.3389/fmolb.2022.1081166

**Published:** 2022-12-15

**Authors:** Allison L. Welter, Yuichi J. Machida

**Affiliations:** ^1^ Mayo Clinic Graduate School of Biomedical Sciences, Mayo Clinic, Rochester, MN, United States; ^2^ Developmental Therapeutics Branch, Center for Cancer Research, National Cancer Institute, Bethesda, MD, United States

**Keywords:** FAM111A, FAM111B, protease, viral replication, Kenny-Caffey Syndrome (KCS), POIKTMP, gracile bone dysplasia, DNA-protein crosslink (DPC)

## Abstract

Proteolysis plays fundamental and regulatory roles in diverse cellular processes. The serine protease FAM111A (FAM111 trypsin-like peptidase A) emerged recently as a protease involved in two seemingly distinct processes: DNA replication and antiviral defense. FAM111A localizes to nascent DNA and plays a role at the DNA replication fork. At the fork, FAM111A is hypothesized to promote DNA replication at DNA-protein crosslinks (DPCs) and protein obstacles. On the other hand, FAM111A has also been identified as a host restriction factor for mutants of SV40 and orthopoxviruses. FAM111A also has a paralog, FAM111B, a serine protease with unknown cellular functions. Furthermore, heterozygous missense mutations in *FAM111A* and *FAM111B* cause distinct genetic disorders. In this review, we discuss possible models that could explain how FAM111A can function as a protease in both DNA replication and antiviral defense. We also review the consequences of *FAM111A* and *FAM111B* mutations and explore possible mechanisms underlying the diseases. Additionally, we propose a possible explanation for what drove the evolution of FAM111 proteins and discuss why some species have two FAM111 proteases. Altogether, studies of FAM111 proteases in DNA repair, antiviral defense, and genetic diseases will help us elucidate their functions and the regulatory mechanisms.

## Introduction

Proteases are essential enzymes required in a myriad of cellular processes such as cell proliferation, differentiation, and cell death. They recognize protein or peptide substrates and cleave amide bonds in a specific and highly regulated manner. Regulation of protease catalytic activity is essential to ensure that protein substrates are proteolyzed at the appropriate time and quantity. Additionally, protease catalytic activity may require small molecules, cofactors, post translational modifications, and even proteolytic cleavage of an inactive zymogen by another protease to ensure enzyme activity is controlled. As key players in biological reactions, proteases are essential across all forms of life, and are found in animals, bacteria, plants, fungi, and viruses.

Based on the mechanism of proteolysis, proteases are classified into six classes: serine, cysteine, threonine, aspartic, glutamic, and metalloproteases. Serine proteases, named after the primary catalytic serine residue, make up approximately one-third of proteases in *Homo sapiens* (Hedstrom, 2002; Rawlings et al., 2018). They contain a catalytic triad, consisting of histidine, aspartate, and serine active site residues, which are all required to hydrolyze the substrate peptide bonds (Blow et al., 1969; Blow, 1997). Despite sharing this conserved catalytic triad, serine proteases vary in their substrate specificity, which is largely defined by the substrate residue N-terminal to the cleavage site (designated as P1). The P1 residue is complementary to the substrate specificity pocket (designated as S1), which differs in size and amino acid composition among various enzymes. For example, chymotrypsin’s S1 pocket is large and favors binding of bulky, aromatic P1 residues, while trypsin has acidic residues in its S1 site to bind basic side chains (Hedstrom, 2002). As substrate specificity can widely vary, a variety of proteases with specific roles are expressed in cells to aid in essential biological reactions.

## FAM111A serine protease

**FIGURE 1:**
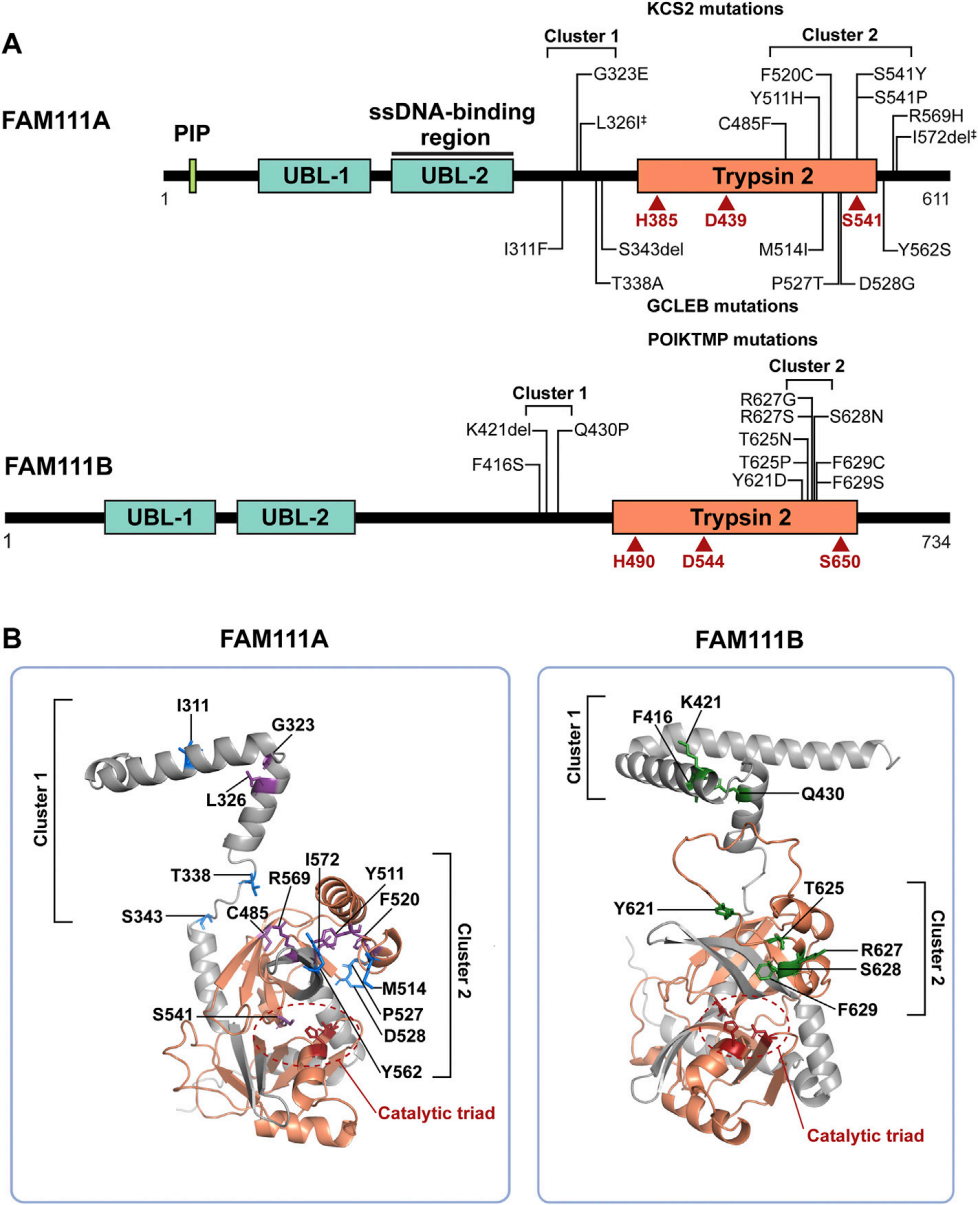
FAM111A and FAM111B protein domain structures and mutations in genetic disorders. **(A)** Schematic representations of FAM111A and FAM111B protein domains. Amino acid mutations in patients for KCS2 (top) and GCLEB (bottom) in FAM111A and POIKTMP in FAM111B are notated. The two regions where patient mutations cluster, the hinge (cluster 1) and enzyme domain (cluster 2), are indicated. Catalytic triads are depicted in red. ^‡^Compound heterozygous mutations. PIP: PCNA-interacting peptide box; UBL-1: Ubiquitin-like domain 1; UBL-2: Ubiquitin-like domain 2; Trypsin 2: Trypsin-like peptidase domain (Pfam ID: PF13365). **(B)** AlphaFold structural prediction of hinge region and enzyme domain of FAM111A (left) and FAM111B (right). Residues mutated in KCS2 (purple), GCLEB (blue) and POIKTMP (green) are indicated. Active site residues are colored in red and circled, and the Trypsin 2 domain is colored in orange. Structures are displayed using PyMOL (http://www.pymol.org/pymol).

FAM111A (FAM111 trypsin-like peptidase A) is a serine protease whose functions in human cells are beginning to emerge. The C-terminus of the human FAM111A contains a Trypsin 2-like serine protease domain (Pfam ID: PF13365) with a conserved catalytic triad (His385, Asp439, Ser541) (Figures 1A,B). The protease activity of FAM111A was recently demonstrated *in vitro* using a purified recombinant protein, which displayed autocleavage activity (Hoffmann et al., 2020). This autocleavage activity was also observed in cells with overexpressed FAM111A, and the prominent cleavage site was determined to be between Phe334 and Gly335 (Kojima et al., 2020). Replacement of the P1 residue, Phe334, with an arginine blocked the autocleavage, suggesting that FAM111A has a chymotrypsin-like specificity. Autocleavage at this site severs the covalent bond between the N-terminal fragment and the enzyme domain; however, it is unknown whether this cleavage occurs with endogenous FAM111A and whether it functions in a manner analogous to zymogen activation seen in other serine proteases. In addition, while the autocleavage activity demonstrates that FAM111A is a protease, it remains to be shown whether FAM111A is capable of directly cleaving proteins other than itself. Identification of potential substrates should facilitate clarification of this point.

The human FAM111A protein contains four main protein domains (Figure 1A): a PCNA interacting peptide (PIP) box (Alabert et al., 2014), two ubiquitin-like domains (UBL-1 and UBL-2) (Rios-Szwed et al., 2020), and a Trypsin 2-like serine protease domain. A PIP box is a short peptide motif that tethers DNA replication and repair factors to PCNA, a sliding clamp for DNA polymerases (Moldovan et al., 2007). The PIP box of FAM111A is required for the localization of FAM111A at DNA replication forks (Alabert et al., 2014), and it was recently shown that endogenous FAM111A protein indeed colocalizes with PCNA foci (Nie et al., 2021). UBL-1 and UBL-2 domains are predicted to have a ubiquitin-like fold structure, as the name suggests. While the exact function of the UBLs is unclear, it was reported that the pair of UBLs resembles the Ras-binding domain of RGS14 (Rios-Szwed et al., 2020), suggesting that the tandem UBLs might be involved in protein-protein interactions. Interestingly, the FAM111A UBL-2 overlaps with the single-strand DNA (ssDNA)-binding region of FAM111A (Kojima et al., 2020), raising a possible role of ssDNA exposed at DNA replication forks in FAM111A regulation. Although cellular functions of FAM111A remain relatively uncharacterized, studies in the past 10 years have implicated FAM111A in antiviral defense, DNA replication, and genetic disorders.

## FAM111A’S role as an antiviral factor

**FIGURE 2:**
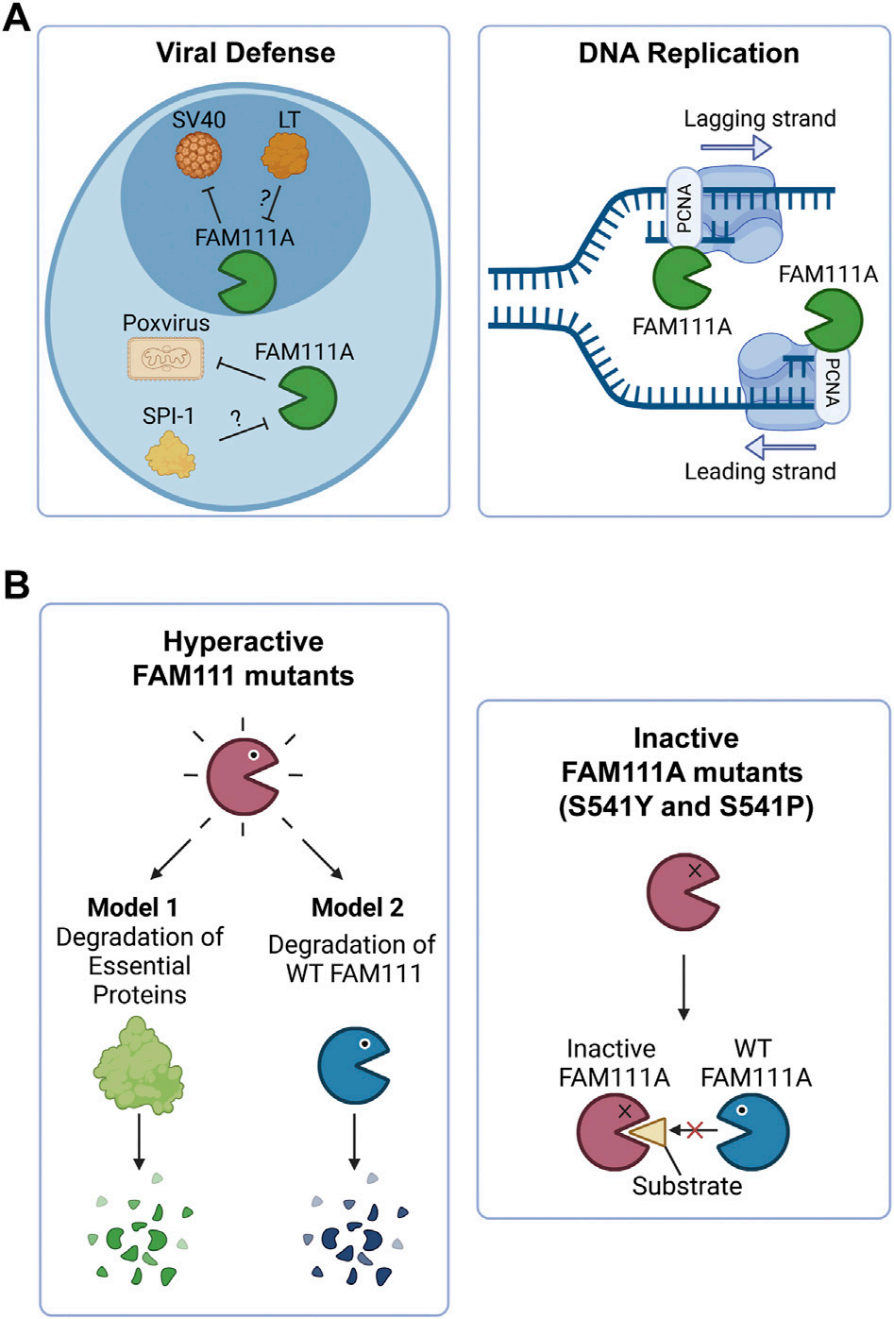
FAM111A’s functions in cells and the implications of FAM111 mutations in human disorders. **(A)** Model of FAM111A’s roles in cells as an antiviral protease and DNA replication fork protein. (Left) FAM111A is a host restriction factor for host range mutants of SV40 and orthopoxviruses. Viral proteins LT (SV40) and SPI-1 (orthopoxvirus) are proposed to inhibit FAM111A protease activity and perturb its antiviral role. (Right) FAM111A functions at the DNA replication fork through its interaction with PCNA. PCNA: proliferating cell nuclear antigen. Figures were created with BioRender.com. **(B)** Possible mechanisms by which heterozygous mutations in *FAM111A* (KCS2/GCLEB) and *FAM111B* (POIKTMP) cause genetic disorders. (Left) Patient associated hyperactive mutants may degrade essential proteins (Model 1) or wild-type FAM111 proteins (Model 2). (Right) Patient associated inactive mutants (S541Y and S541P) of FAM111A may interfere with functions of wild-type enzymes by sequestering substrates. Figures were created with BioRender.com.

The FAM111A protein was first identified as a host restriction factor for the host range mutants of the polyomavirus SV40, which is an oncogenic DNA virus (Figure 2A, left) (Fine et al., 2012). This study demonstrated that FAM111A and SV40 Large T antigen (LT) interact through the serine protease domain of FAM111A and the C-terminal host range domain of LT, which was truncated in the host range mutant viruses (Fine et al., 2012). During SV40 infections, either knockdown of FAM111A or expression of the SV40 LT C-terminus in trans was sufficient for supporting replication of the SV40 host cell mutants in non-permissive cells (Poulin and DeCaprio, 2006; Fine et al., 2012). These findings are consistent with the idea that LT inhibits FAM111A protease activity and perturbs its host restriction role during SV40 infection (Fine et al., 2012). With this role, FAM111A appears to function through an antiviral defense mechanism, whereas the SV40 virus has developed a strategy to antagonize this attack through the LT C-terminus. What does FAM111A target in this antiviral mechanism? During SV40 infections, FAM111A localizes to viral replication centers (Tarnita et al., 2019). Depletion of FAM111A in infected cells results in higher viral replication center numbers (Tarnita et al., 2019), suggesting that FAM111A might proteolyze a key factor necessary for the viral replication process. Additionally, FAM111A disrupts nuclear permeability during SV40 infection, and nuclear pore complex proteins were identified as putative FAM111A targets (Nie et al., 2021). As a host restriction factor, FAM111A may target nuclear pore proteins, as well as viral proteins, to impede viral replication.

The antiviral function of FAM111A was also demonstrated with another DNA virus, orthopoxvirus (Panda et al., 2017). In this study, knockdown of FAM111A, RFC3, or IRF2 were found to allow replication of host range mutants of orthopoxviruses in non-permissive cells. Because the host range mutants used in this study lack the viral serine protease inhibitor gene, SPI-1, this provides genetic evidence supporting that SPI-1 inhibits FAM111A protease activity to promote viral replication, disrupting its role as an antiviral factor. While IRF2 turned out to be required for FAM111A expression, identification of RFC3 as a host restriction factor provides important insight. RFC3 is a subunit of the Replication Factor C (RFC) complex necessary for the clamp loading of PCNA onto DNA (Majka and Burgers, 2004). Given that FAM111A interacts with PCNA at DNA replication sites, it is tempting to think that FAM111A requires PCNA loaded by RFC to function as an antiviral factor.

Altogether, a common pattern of interactions between a host cell and viruses emerges: FAM111A protease activity is employed by host cells to restrict viral replication while viruses evolve means to counterattack FAM111A (i.e., LT in SV40 and SPI-1 in orthopoxviruses). A recent study suggested that FAM111A, RFC3, and IRF2 also restrict replication of Zika virus (ZIKV) (a single-stranded RNA virus) (Ren et al., 2021), suggesting that the effect of FAM111A-dependent antiviral mechanisms could reach beyond DNA viruses. It would be interesting to explore whether ZIKV encodes an anti-FAM111A factor.

## FAM111A at the replication fork

FAM111A was identified in proteomics studies as one of the uncharacterized proteins enriched at nascent DNA (Alabert et al., 2014; Wessel et al., 2019). FAM111A utilizes its PIP box to localize to replication forks through its interaction with PCNA (Alabert et al., 2014) (Figure 2A, right). It was proposed that FAM111A facilitates PCNA loading on chromatin, as FAM111A knockdown resulted in reduced levels of PCNA on chromatin and impaired DNA replication (Alabert et al., 2014). Whether the protease activity of FAM111A is required for this function remains unknown.

New insight into the role of FAM111A protease activity at DNA replication forks was obtained in a study investigating involvement of FAM111A at stalled replication forks (Kojima et al., 2020). The study of Kojima et al. showed that loss of FAM111A leads to spontaneous accumulation of topoisomerase 1 cleavage complexes (TOP1ccs) (Kojima et al., 2020), in which TOP1 is trapped in a stable reaction intermediate complex with the active site tyrosine crosslinked to 3′-end of the DNA backbone due to nearby DNA damage (Pourquier and Pommier, 2001). TOP1ccs are a type of DNA-protein crosslink (DPC), which are bulky protein obstacles that block the replication fork machinery from progressing, thereby threatening genomic integrity and impairing cell survival (Stingele and Jentsch, 2015). TOP1ccs can be stabilized exogenously through the TOP1 inhibitor, camptothecin (CPT) (Pommier, 2006). Upon stabilization of TOP1ccs with CPT, *FAM111A* knockout (KO) cells have decreased cell survival and increased replication fork stalling, suggesting that FAM111A is essential for cell survival and for promoting replication when forks are stalled by stabilized TOP1ccs (Kojima et al., 2020). *FAM111A* KO cells also exhibit stalled replication forks and display hypersensitivity to poly (ADP-ribose) polymerase inhibitors (PARPis), which trap PARP enzymes at ssDNA breaks to form DPC-like tight DNA-protein complexes (Murai et al., 2012; Murai et al., 2014). Importantly, an active site mutant of FAM111A (S541A) failed to rescue the replication defects in the presence of CPT or PARPis, as well as spontaneous accumulation of TOP1ccs in *FAM111A* KO cells (Kojima et al., 2020), suggesting that FAM111A protease activity is necessary for this function. Based on these observations, stabilized TOP1ccs, trapped PARPs, and possibly other protein obstacles, DPCs, or replication fork proteins are proposed substrate candidates for the FAM111A serine protease.

In addition to its enzyme activity, the PIP box is important for FAM111A to promote DNA replication at TOP1ccs. This is perhaps not surprising, as the PIP box is necessary for the recruitment of FAM111A to the sites of DNA replication. What remains unclear is whether FAM111A is an integral component of the replication machinery or is instead recruited to replication forks in response to fork stalling. Colocalization of endogenous FAM111A with PCNA foci (Nie et al., 2021) might support the first possibility, yet it remains possible that fork stalling at protein obstacles might be common in cells and require frequent engagement of FAM111A during the normal replication process.

The metalloprotease, SPRTN, is the first protease demonstrated to proteolyse DPCs that block replication progression in mammalian cells (Lopez-Mosqueda et al., 2016; Stingele et al., 2016; Vaz et al., 2016; Morocz et al., 2017; Larsen et al., 2019). KO of *Sprtn* in mice causes embryonic lethality, and conditional KO in mouse embryonic fibroblasts causes Top1cc accumulation, DNA replication defects, and eventually cell death (Maskey et al., 2014; Maskey et al., 2017). In contrast, *Fam111a* KO mice are viable without an overt phenotype (Ilenwabor et al., 2022), and *FAM111A* KO is well-tolerated at the cellular level (Hoffmann et al., 2020; Kojima et al., 2020). Therefore, SPRTN appears to be more important in terms of viability. However, *FAM111A* KO cells exhibit loss of viability and profound replication fork defects in the presence of DPC-inducing agents, suggesting that FAM111A might become critical with excessive levels of DPCs (Kojima et al., 2020). Better understanding of the relationship between FAM111A and SPRTN would facilitate illuminating the exact role of FAM111A at replication forks.

## *FAM111A* mutations in genetic diseases

Mutations in the *FAM111A* gene were found to be the primary cause of Kenny-Caffey Syndrome type 2 (KCS2) and the more severe disorder Gracile Bone Dysplasia (GCLEB), also known as osteocraniostenosis (Figure 1A) (Kenny and Linarelli, 1966; Caffey, 1967; Unger et al., 2013; Guo et al., 2014; Isojima et al., 2014; Nikkel et al., 2014; Kim et al., 2015; Abraham et al., 2017; Wang et al., 2019; Cavole et al., 2020; Deconte et al., 2020; Pemberton et al., 2020; Turner et al., 2020; Cheng et al., 2021; Eren et al., 2021; Lang et al., 2021; Muller et al., 2021; Yerawar et al., 2021; Rosato et al., 2022). In both diseases, patients present with stenosis and thickening of long bones, hypoparathyroidism, hypocalcemia, and short stature. Currently, there are 24 reported KCS2 and 10 GCLEB cases with confirmed *FAM111A* mutations. Heterozygous *de novo* mutations are most common, except for one maternally inherited case (Nikkel et al., 2014) and one inherited compound heterozygous case (Eren et al., 2021). These mutations are missense mutations and are clustered in two regions. The AlphaFold predicted structure of FAM111A shows the first cluster of residues mutated in disease are located between the UBLs and the Trypsin 2 domain in a flexible hinge region, while the second cluster falls within the enzyme domain (Figure 1B).

Recent studies demonstrated that most patient associated *FAM111A* mutations are gain-of-function mutations that lead to hyperactivity of the protease (Hoffmann et al., 2020; Kojima et al., 2020; Nie et al., 2021). Ectopic overexpression of the patient associated FAM111A mutants causes reduced DNA synthesis, disruption of nuclear structure, and cell death due to dysregulated protease activity of FAM111A (Hoffmann et al., 2020; Nie et al., 2021). Therefore, degradation of essential proteins by dysregulated FAM111A could be an underlying mechanism of the diseases (Figure 2B, left panel, Model 1). However, this model does not explain three cases of KCS2 in which the active site serine was mutated to tyrosine (S541Y) (Abraham et al., 2017) or proline (S541P) (Cheng et al., 2021), as these mutations are expected to inactivate the enzyme. This suggests that both hyperactivation and inactivation of FAM111A have the same consequences and cause KCS2. One possible explanation for this paradox could be that the hyperactive mutants of FAM111A might degrade wild-type FAM111A protein, resulting in its depletion (Figure 2B, left panel, Model 2). This model would be able to explain the effects of hyperactivating mutants by a loss of function mechanism. However, it is still unclear how inactivating FAM111A mutants (S541Y and S541P) can cause disease in the presence of a FAM111A wild-type allele. One possibility is that the inactive enzyme might trap and sequester substrates from the wild-type FAM111A protein, interfering with its function (Figure 2B, right panel). Taken together, it is possible that loss of wild-type FAM111A protein (or activity) might be the primary cause of KCS2 and GCLEB. Better models that can examine the effect of heterozygous mutations in the *FAM111A* gene are required to fully understand the underlying mechanism of the diseases.

## FAM111B: A paralog of FAM111A

The story of FAM111A goes beyond the *FAM111A* gene, as it has a paralog, *FAM111B* (FAM111 trypsin-like peptidase B). *FAM111A* and *FAM111B* are adjacent on chromosome 11, suggesting that *FAM111B* likely arose from a gene duplication event. FAM111B has a conserved serine protease domain (46% sequence similarity with FAM111A) with an intact catalytic triad (His490, Asp544, and Ser650) and two N-terminal UBLs (Figure 1A). Despite lacking a PIP box, FAM111B is enriched at nascent chromatin (Rios-Szwed et al., 2020), implying that it may play a similar role to FAM111A at the fork. Indeed, FAM111B was found among FAM111A-associated proteins in proteomics studies (Hoffmann et al., 2020; Rios-Szwed et al., 2020), and the interaction in cells was further suggested by co-immunoprecipitation of FAM111A and FAM111B from cells that overexpressed both proteins (Hoffmann et al., 2020). In contrast to FAM111A, FAM111B does not interact with SV40 LT and has not been implicated in cellular protection from SV40 (Fine et al., 2012). However, a recent study showed that FAM111B is upregulated after infection with human adenovirus and functions as a host restriction factor (Ip et al., 2021), suggesting that FAM111B might be an antiviral factor with different specificity.

## FAM111B in diseases

Mutations in the *FAM111B* gene also lead to a genetic disorder, but the phenotypes are distinct from disorders with *FAM111A* mutations. Autosomal dominant mutations found in the *FAM111B* gene are the primary cause of hereditary fibrosing poikiloderma with tendon contractures, myopathy, and pulmonary fibrosis (POIKTMP), a rare disorder with 37 confirmed cases (Figure 1A) (Khumalo et al., 2006; Mercier et al., 2013; Mercier et al., 2015; Seo et al., 2016; Goussot et al., 2017; Takeichi et al., 2017; Chen et al., 2019; Zhang et al., 2019; Dokic et al., 2020; Roversi et al., 2021; Hoeger et al., 2022; Macchiaiolo et al., 2022; Takimoto-Sato et al., 2022; Wu et al., 2022). Like the disease-associated mutations in *FAM111A*, POIKTMP mutations within *FAM111B* cluster within the hinge (cluster 1) or the enzyme domain (cluster 2) (Figure 1B). Interestingly, mutations within the protease domain have more severe disease phenotypes than within the hinge (Arowolo et al., 2022). The main symptoms of POIKTMP include but are not limited to irregular skin pigmentation and atrophy (poikiloderma), advancing muscle weakness of all four limbs, tendon contractures, pulmonary fibrosis, and pancreatic dysfunction. In addition, two cases of fatal pancreatic cancer have been reported in POIKTMP patients (Goussot et al., 2017; Mercier et al., 2019). This implies that *FAM111B* mutations not only cause the multisystemic phenotypes observed in POIKTMP patients, but also might promote cancer formation. Disease associated mutations in FAM111B cause hyperactivation, suggesting that the phenotypes observed in this disorder might arise from dysregulated protease activity (Hoffmann et al., 2020) (Figure 2B, left panel, Model 1). Alternatively, the disease might be caused by a loss of FAM111B protein if its hyperactivation results in autocleavage and depletion of FAM111B (Figure 2B, left panel, Model 2), as discussed above for disease-associated *FAM111A* mutations.

FAM111B overexpression is connected to poor prognosis and progression in cancers. High expression of FAM111B is correlated with poor outcome in breast cancer, and FAM111B was suggested to be important for cell proliferation, migration, and invasion in breast cancer (Li et al., 2022). In lung adenocarcinoma, FAM111B expression also correlates with poor outcome (Sun et al., 2019; Kawasaki et al., 2020). Here, FAM111B was reported to be a direct target of p53 (Sun et al., 2019) and suggested to promote cell cycle progression through p16 degradation (Kawasaki et al., 2020). Altogether, the correlation of high FAM111B with poor survival in several cancer types suggests that it may be an oncogene. This might be consistent with the increased cancer susceptibility observed in POIKTMP patients, if hyperactivation of FAM111B exhibits gain-of-function effects (Hoffmann et al., 2020).

## Evolution of FAM111 proteases

**FIGURE 3:**
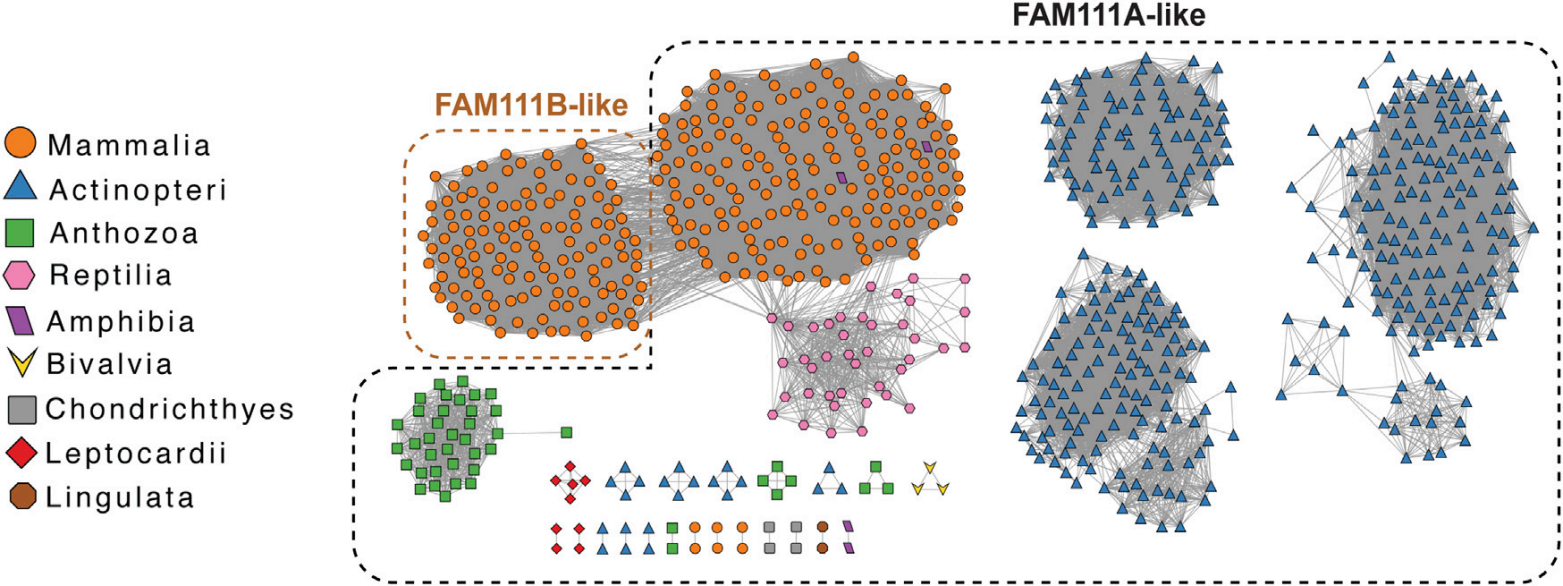
FAM111A ortholog sequence similarity network with FAM111B paralogs. The sequence similarity network (SSN) was generated using the Enzyme Function Initiative’s Enzyme Similarity Tool (https://efi.igb.illinois.edu/efi-est), using *H. sapiens* FAM111A protein as the query sequence and displayed with Cytoscape (https://cytoscape.org). All nodes from initial results are displayed, and only edges with 47–100% sequence identity between nodes are shown. Nodes are annotated by biological class, and nodes containing putative FAM111B paralogs are notated.

**FIGURE 4:**
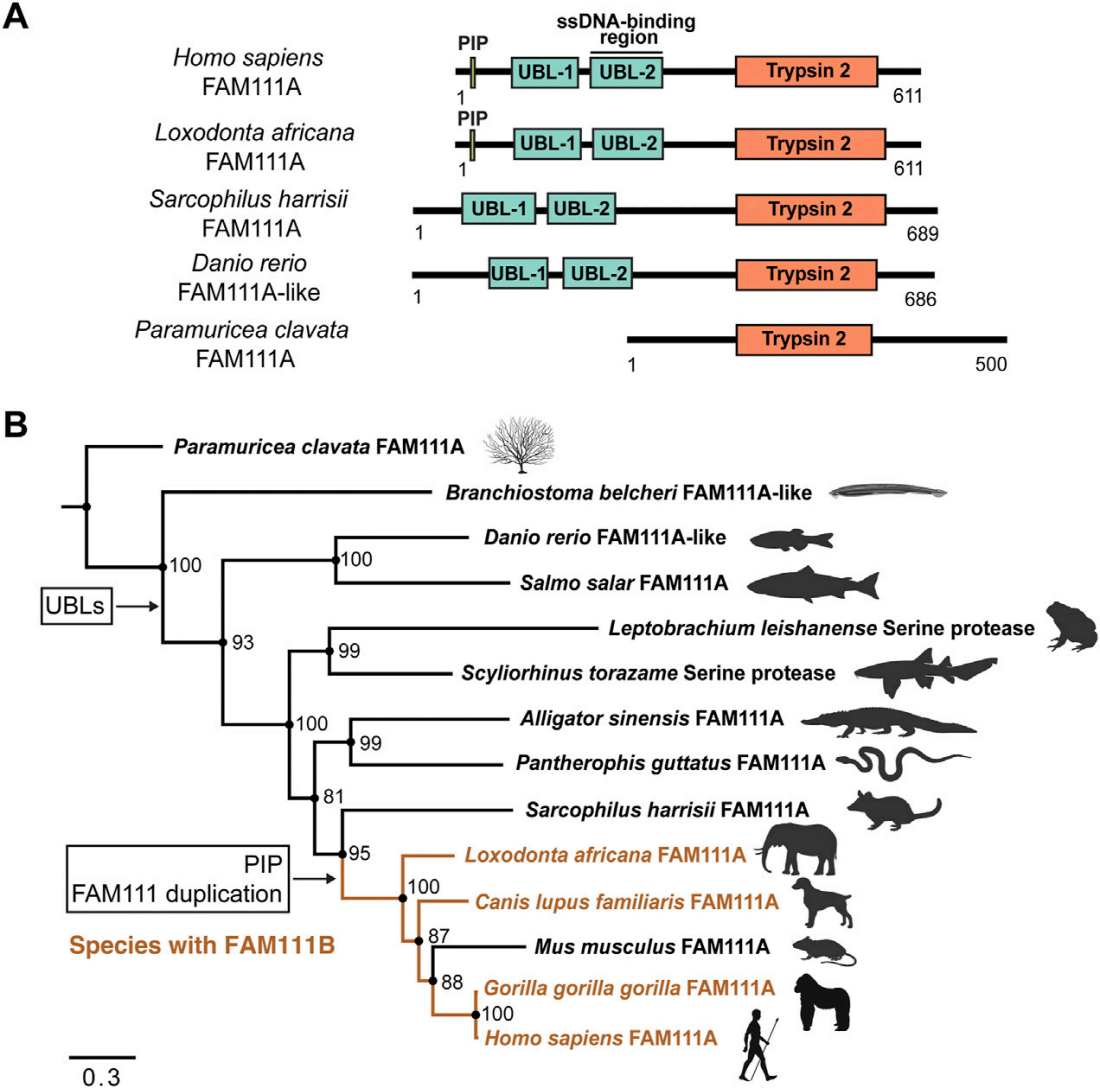
Phylogenetic inference of FAM111A orthologs. **(A)** A schematic representation of domain structures for representative orthologs of FAM111A. PIP: PCNA-interacting peptide box; UBL-1: Ubiquitin-like domain 1; UBL-2: Ubiquitin-like domain 2; Trypsin 2: Trypsin-like peptidase domain (Pfam ID: PF13365). **(B)** Bayesian phylogenetic prediction of FAM111A orthologs. A Bayesian phylogenetic tree inferred by MrBayes (http://nbisweden.github.io/MrBayes) was generated from a CLUSTAL Omega (https://www.ebi.ac.uk/Tools/msa/clustalo) FAM111A sequence alignment containing 14 representative species, selected from larger scale multiple sequence alignments and trees. Proteins included in analysis are *Paramuricea clavata* FAM111A (UniProt ID: A0A7D9LSX1)*, Branchiostoma belcheri* FAM111A-like (UniProt ID: A0A6P4ZC58), *Danio rerio* FAM111A-like (UniProt ID: A0A8M2BD64), *Salmo salar* FAM111A (UniProt ID: A0A1S3N1P2), *Leptobrachium leishanense* Serine protease (A0A8C5WH67), *Scyliorhinus torazame* Serine protease (A0A401QCT3), *Alligator sinensis* FAM111A (A0A1U8DS27), *Pantherophis guttatus* FAM111A (A0A6P9C8G8), *Sarcophilus harrisii* FAM111A (G3VJT4), *Loxodonta africana* FAM111A (G3T8F5), *Canis lupus familiaris* FAM111A (A0A8C0SC36), *Mus musculus* FAM111A (Q9D2L9), *Gorilla gorilla gorilla* FAM111A (G3RXJ5), and *Homo sapiens* FAM111A (Q96PZ2). Among-site rate variation was set to inverse gamma and the outgroup was defined as *Paramuricea clavata* (determined as outgroup from prior tree analyses). Analysis ran until the average standard deviation of split frequencies approaches zero (<0.01). Node support values are Bayesian inference posterior probabilities, written as percentages. Species which contain FAM111B are highlighted in orange. The points where the PIP box and UBLs appear, as well as the occurrence of the FAM111 gene duplication are indicated. Scale bar indicates estimated substitutions per site. Figure was created with FigTree (http://tree.bio.ed.ac.uk/software/figtree) and BioRender.com.

How well are FAM111 proteases conserved among species and how did they evolve? A sequence similarity network (SSN) (Zallot et al., 2019) using the *H. sapiens* FAM111A protein as the query sequence retrieved 866 sequences from 249 species and nine biological classes within Animalia (Figure 3). In addition to identifying putative FAM111A orthologs, this analysis also found FAM111B paralogs within the network. The phylogenetic inference of a Bayesian phylogenetic tree (Ronquist et al., 2012; Nascimento et al., 2017) consisting of 14 putative FAM111A orthologs (Figure 4A) provides insight into the evolution of FAM111 proteases and predict when its various functions evolved (Figure 4B).

All the species in the phylogenetic analysis contain a Pfam predicted Trypsin 2 domain, with an intact catalytic triad. The Trypsin 2 domain is the highest conserved domain within FAM111A orthologs, which is expected since it is catalytic domain.

Within the tree, the two N-terminal UBL domains are conserved in all species, except the marine invertebrates *Paramuricea clavata* (violescent sea-whip; soft coral) FAM111A (UniProt ID: A0A7D9LSX1) and *Branchiostoma belcheri* (lancelet) FAM111A-like (UniProt ID: A0A6P4ZC58) proteins. This suggests that the UBLs evolved at a point between marine invertebrates and vertebrates. No FAM111A orthologs were found to have only one UBL; instead, the majority of species tend have two UBLs or none.

The PIP box is first observed within the Mammalia clade. However, the PIP box is not present in early mammals within the orders Marsupials and Monotremes. Proteins from species in these orders without a PIP box include *Sarcophilus harrisii* (Tasmanian devil) FAM111A (UniProt ID: G3VJT4) and *Ornithorhynchus anatinus* (platypus) FAM111A (UniProt ID: F7AAR7). This supports that the PIP box in FAM111A evolved within Mammalia, following the Marsupial branch (Deakin et al., 2012). Overall, FAM111A’s Trypsin 2 domain and UBLs are conserved in many orthologs, while the PIP box more recently evolved within Mammalia.

Interestingly, the gene duplication event between *FAM111A* and *FAM111B* coincides with the appearance of the PIP box in FAM111A within Mammalia. In the phylogenetic analysis, *S. harrisii* branched out earliest in mammals before the gene duplication, and none of the species within other classes have *FAM111B*. This suggests that the gene duplication event between *FAM111A* and *FAM111B* also occurred after Marsupial evolution within Mammalia. As we do not observe a PIP box in FAM111B orthologs, it is likely the PIP box emerged after the gene duplication event. Therefore, it is possible that the FAM111 gene duplication granted the opportunity for the emergence of the PIP box in FAM111A, since FAM111A localization can then be restricted to the replication fork.

## Putative FAM111A orthologs and related serine proteases in lower organisms

The SSN of *H. sapiens* FAM111A also identified its possible orthologs in lower organisms. Two of the sequences included in the phylogenetic analysis, from classes Amphibia [*Leptobrachium leishanense*; Leishan spiny toad (UniProt ID: A0A8C5WH67)] and Chondrichthyes [*Scyliorhinus torazame*; cloudy catshark (UniProt ID: A0A401QCT3)] were annotated as “Serine proteases” and not as FAM111A in UniProt. AlphaFold structural prediction (Jumper et al., 2021), alongside sequence alignment with other orthologs, found both proteases contain two N-terminal UBLs and shared homology to *H. sapiens* FAM111A, suggesting that these two serine proteases are likely FAM111A-like serine proteases. It is possible that FAM111A has more distant orthologs than identified in our SSN, but those orthologs without UBL domains would require further functional annotations to be classified as a FAM111A ortholog.

Based on the sequence alignments of the enzyme domain, FAM111A and FAM111B are classified in the S1 family (chymotrypsin peptidases) of the clan PA in the peptidase database, MEROPS (Rawlings et al., 2008; Rawlings et al., 2018). Within the S1 family, FAM111A and FAM111B are two of four peptidases unassigned to a subfamily out of the 704 entries. Many proteases within the S1 family are extracellular, and FAM111A and FAM111B are two of the few intracellular proteases in the family, another example being HrtA2. In a search for structural homologs to FAM111 proteases using SWISS-MODEL (Waterhouse et al., 2018), members of peptidase subfamily S1C (DegP peptidase), which include the HtrA and Deg proteases, are the top candidates. Notably, the Deg proteases are involved in stress responses (Huesgen et al., 2005), therefore the functions of FAM111A in antiviral defense and replication stress response are in line with the functions of the peptidases in this family. In addition, Deg family peptidases contain PDZ regulatory domains (Ponting, 1997), thus FAM111A might have evolved its own regulatory domain consisting of two UBLs. To assess whether the FAM111 peptidases belong to the subfamily S1C or a novel subfamily, future studies must address their substrate specificities and reevaluate their classification as more FAM111A orthologs become annotated. Although FAM111A and FAM111B are relatively uncharacterized from a functional standpoint, the two proteases might fit into their own novel subfamily, as they have unique features compared to other members of the S1 family and S1C subfamily.

## Relationship of FAM111A and FAM111B

The gene duplication event between *FAM111A* and *FAM111B* likely occurred within Mammalia. The phylogenetic analysis highlighted that the gene duplication event is predicted to have occurred following Marsupial evolution; however, species that branch out later within Mammalia do not always contain both proteases. The SSN identified a small subset of species within the biological order Artiodactyla (even-toed ungulates and whales) which only have FAM111B, and not the *FAM111A* gene. These include *Physeter catodon* (sperm whale) FAM111B (UniProt ID: A0A2Y9TIY4), *Ovis aries* (sheep) FAM111B (UniProt ID: A0A6P3T9Q5), and *Capra hircus* (goat) FAM111B (UniProt ID: A0A452ENK6). We observe a similar situation with *Mus musculus* (mouse), which only has *Fam111a* (UniProt ID: Q9D2L9) but is predicted to have evolved after the duplication event. This demonstrates either certain species only require one FAM111 protease, or FAM111A and FAM111B have semi-redundant functions and both are not required for survival. Interestingly, *H. sapiens* FAM111A and FAM111B were found to interact with each other by FAM111A interactome studies and co-immunoprecipitation (Hoffmann et al., 2020; Rios-Szwed et al., 2020). These observations bring up interesting questions on whether FAM111A and FAM111B work together, why certain species need two FAM111 proteases, whether FAM111B has similar roles in fork protection and antiviral defense mechanisms like FAM111A.

## What drove the evolution of FAM111 proteases?

*Fam111a* KO mice are viable with no overt phenotypes (Ilenwabor et al., 2022). This suggests that *Fam111a* is not an essential enzyme in mouse, and that retention of FAM111A in species may be due to selective pressure for its other roles, such as viral defense. FAM111A’s Trypsin 2 protease domain is the most evolutionary conserved out of four currently identified domains. The PIP box, however, is predicted as the most recently evolved domain within Mammalia. Because this motif is required for FAM111A localization to the replication fork, its recent development implies either FAM111A is recruited to replication sites in distant orthologs without a defined PIP box, or it is not a replication fork protease in those species. If the latter is true, one possible explanation is that FAM111A protease activity originally had other roles in cells, and its protease activity was recently repurposed to cleave TOP1ccs and possibly other DPCs that stall replication forks. Because FAM111A is a host restriction factor, this could have been its initial main function until it evolved to be important during replication stress.

## Perspectives

Considering what is currently known about FAM111A as a host restriction factor and protease that promotes DNA replication at protein obstacles, we can speculate about a potential mechanism that allows FAM111A to function as an antiviral protease as well as a putative DNA repair factor. Because both FAM111A and RFC3 are host restriction factors, we postulate that FAM111A is recruited to replicating viral genomes through a mechanism involving PCNA loaded by RFCs. Because FAM111A is an active protease, it is plausible that FAM111A might cleave viral proteins to impede viral replication. As FAM111A is hypothesized to be a DPC protease, it would make more sense if the viral protein targeted by FAM111A is degraded when it forms a DPC. This model predicts that viral replication has a process that involves a DPC or a DPC-like tight DNA-protein complex that is programed to form as an essential step. In fact, it was recently reported that maintenance of the Epstein-Barr virus episome involves a DPC containing the viral protein EBNA1 (Dheekollu et al., 2021). Therefore, if viruses rely on a programed DPC for their propagation, FAM111A may proteolyse these essential complexes and disrupt viral replication. Future studies on FAM111A’s role as a host restriction factor will provide mechanistic insight into defense mechanisms human cells use to fight viral infections. This new knowledge in turn could shed new light on how FAM111A facilitates DNA replication at protein obstacles formed on the host genome.

We also anticipate future research on the FAM111 proteases will include mechanistic studies examining how patient associated mutations in *FAM111A* and *FAM111B* cause hyperactivation of the proteases. In these genetic disorders, patient mutations in *FAM111A* and *FAM111B* cluster in two regions: 1) a hinge region between the UBLs and the Trypsin 2 domain and 2) within the Trypsin 2 domain. As the UBLs in FAM111 proteases might have a regulatory role, it is possible that mutations in this hinge region may disrupt the UBLs’ ability to regulate the enzyme domain. The second cluster of mutations found within the Trypsin 2 domain might alter the enzyme conformation or possible intramolecular interactions with the UBLs. In addition, it will be important to assess whether KCS2/GCLEB and POIKTMP are caused by degradation of essential proteins by hyperactive FAM111 proteases or a lack of the FAM111 proteases (or activity), as such information is crucial for determining whether inhibition of FAM111 proteases is a valid therapeutic strategy.
